# Evaluating a pilot community-based FITMIND exercise programme for psychosis in Hong Kong

**DOI:** 10.1186/s12888-023-04901-x

**Published:** 2023-05-31

**Authors:** Yi Nam Suen, Edwin Ho Ming Lee, Christina Oi Bun Lam, Christy Lai Ming Hui, Sherry Kit Wa Chan, Wing Chung Chang, Eric Yu Hai Chen

**Affiliations:** 1 Department of Psychiatry, Li Ka Shing Faculty of Medicine, The University of Hong Kong, Hong Kong, China; 2 The State Key Laboratory of Brain and Cognitive Sciences, The University of Hong Kong, Hong Kong, China

**Keywords:** FITMIND, psychosis, community, pilot trial

## Abstract

**Background:**

Exercise interventions can improve clinical symptoms and cognition in patients with psychosis in addition to their physical health. However, their benefits may not be maximally generalised to those who cannot access gymnasium facilities, which were commonly required previously. This study evaluated a 12-week community exercise programme named FITMIND, which aims to help patients with psychosis establish exercise habits through easy-to-learn aerobic exercise and yoga, with the support of trained volunteers.

**Method:**

This study analysed the profiles of 49 patients with psychosis who were referred by the case manager of the early psychosis programme in the public hospital in Hong Kong or enrolled in the programme through the project website. The outcome measures were working memory, physical activity (PA) participation, quality of life, and mood symptoms.

**Results:**

At baseline, seven participants (14.3%) met the recommendation of the PA for severe mental illnesses. After the 12-week programme, participants demonstrated significant improvement in vigorous-intensity PA, moderate-to-vigorous PA, compliance with international guidelines for PA, and mood symptoms.

**Conclusion:**

The FITMIND exercise programme is a feasible community-based intervention that can improve PA participation and mood in patients with psychosis. Further systematic studies are needed to examine the long-term beneficial effects of the programme.

**Supplementary Information:**

The online version contains supplementary material available at 10.1186/s12888-023-04901-x.

## Background

Individuals with psychosis have a lower life expectancy, reflected in a higher overall mortality rate than the general population [1, 2]. Specifically, those with schizophrenia lose an average of 28.5 years of life expectancy [3]. While the effectiveness of antipsychotic medications in improving positive symptoms is well established [4], the findings were less robust for negative symptoms and cognitive impairment, but they are more important for overall functioning. Therefore, to complement the insufficiency of medication treatment alone for rehabilitation, adjunctive psychosocial interventions are needed [5].

Exercise interventions, such as aerobic exercise and yoga, can be effective in improving not only a patient’s clinical symptoms [6, 7], but also cognitive functioning [8–13], interpersonal, and functional outcomes [14]. Regarding the improvement in cognitive function, several studies suggested that not only the type and duration of the exercise matter, but also the amount and intensity [15–17]. Particularly, previous studies found that aerobic exercise and yoga, in conjunction with an increase in hippocampal volume [18], were associated with improvements in both working memory and depressive symptoms, and yoga can additionally improve verbal acquisition and attention [19]. Although a recent study found that a dose-response relationship is not linear, high-dose exercise was found to be associated with greater improvement in cognitive functioning [20]. Despite numerous benefits, many patients with psychosis have poor self-motivation, little social support, cognitive deficits, and self-stigma [21–23], which may hinder their participation in exercise.

Indeed, most of the exercise interventions were gymnasium-based, which may make it difficult to generalise the benefits to community members who do not have access to the facilities. To facilitate the uptake of exercise interventions in patients with psychosis, making them available in the community may be a possible solution [24], especially if the programme design involves community members and academic researchers [24–26]. To facilitate participation, the community mental health service should address the high level of stigma and strengthen the social support aspects [27]. The community approach has been used in exercise interventions in various populations [28–30] but has not been adequately studied in psychosis populations. Therefore, in this study, a community-based exercise programme named FITMIND was developed and tested among patients with psychosis in Hong Kong.

In this study, we hypothesised that implementing an exercise programme tailored for patients with psychosis in the community would be feasible and that participants who completed the exercise programme would demonstrate a significant improvement in their physical activity (PA) participation, overall wellbeing, mood symptoms, and cognitive functioning.

## Methods

### Study setting

This study analysed data from 49 out of 87 participants who participated in the FITMIND exercise programme and provided post-intervention data from March 2018 to June 2021 (i.e., 38 participants were excluded since they had not provided the post-intervention data). Participants were patients who received a primary diagnosis of schizophrenia, schizoaffective disorder, schizophreniform disorder, brief psychotic disorder, or other specified psychotic disorder at any public hospital in Hong Kong. They were either referred by the case manager of the Early Assessment Service for Young People with Early Psychosis (EASY service) in Hong Kong or self-referred by applying for participation on the programme’s website or contacting programme staff by phone or email (http://www.episo.org/fitmind10/). The EASY service is a specialised service for patients with psychosis featuring public education and multidisciplinary early intervention [31]. Aiming to become a widely accepted, non-stigmatising community service, there were no specific inclusion or exclusion criteria for participation in the FITMIND exercise programme as long as the participants provided certified proof of a diagnosis of psychosis from their psychiatrist. However, the certified yoga teacher would assess the patient’s suitability to practise yoga and participate in the group. For participation in the current evaluation study, the participants were required to provide informed written consent.

### FITMIND exercise programme

The FITMIND exercise programme is a free-of-charge programme consisting of twelve weekly 90-minute sessions of FITMIND 10 and FITMIND Yoga 23, organised by the Early Psychosis Foundation and introduced in 2013 in Hong Kong [32]. The programme is offered every weekend at four locations in Hong Kong, hosted by trained, certificated external instructors and supported by trained volunteers. Volunteers also help conduct assessments, help participants set goals for exercise each week, and review their progress. *FITMIND 10* consists of ten formulas of static and dynamic exercises designed for patients with psychosis [33]. *FITMIND Yoga 23* consists of 23 formulas of yoga postures designed for patients with psychosis [34]. These exercises and postures are “easy-to-learn” and are designed to facilitate patients with psychosis in developing an exercise habit to improve their cognitive functioning more effectively as an adjunct to medication. The group format and volunteer involvement are intended to enhance peer and social support and provide patients with a sense of acceptance in the community. and social support and provide patients with a sense of acceptance in the community.

### Data collection

Data collected included demographic information (age, gender, years of education, and living condition), physical fitness (resting heart rate, resting blood pressure, body mass index [BMI] and waist-to-hip ratio [WHR], cognitive function, physical activity engagement, status of well-being, and experience of mood symptoms) at the beginning of the first session of the programme by trained volunteers who were supervised by the psychiatrists in the team. Cognitive functions, physical activity, quality of life, and mood symptoms were also assessed after the programme. The pre- and post-intervention assessments could be conducted by the same or separate volunteers, depending on the manpower arrangement of the session.

#### Physical fitness

The resting heart rate and blood pressure (BP) were measured using a portable blood pressure monitor. The normal range of the two measures is 60–80 bpm and ≤ 130 mmHg for systolic BP [SBP] and ≤ 80 mmHg for diastolic BP [DBP] [35]. Participants’ height, waist, and hip circumferences were measured using a soft ruler, and their weight was measured using a portable weight scale. A BMI > 23 is considered overweight [36] and a WHR > 0.90 for men and > 0.85 for women is signified as central obesity in the Asian population [37].

#### Cognitive function

The participant’s cognitive function was measured particularly in the domain of working memory using the digit span subset (Wechsler Adult Intelligence Scale-Revised, WAIS-R-HK) [38]. Only working memory was chosen, as it was consistently found to be improved through exercise interventions. Moreover, only one domain was chosen as the programme was conducted in a community setting, so a long and complicated assessment would not be suitable.

#### Physical activity level

We assessed participants’ physical activity (PA) level in the past week using the Chinese version of the International Physical Activity Questionnaire Short Form (IPAQ-C-SF) [39, 40]. The seven-item IPAQ-C-SF records self-reported physical activity in the last seven days. According to the IPAQ scoring protocol, total minutes spent on walking, moderate-, and vigorous-intensity physical activity (MPA and VPA, respectively) over the last seven days were multiplied by 3.3, 4.0, and 8.0 to yield the metabolic equivalent task minutes per week (MET-min/week) [41]. The level of moderate-to-vigorous intensity PA (MVPA) is calculated by summing the MPA and VPA. According to the recommendation by the European Psychiatric Association, patients with severe mental illness should achieve 900 MET-min of MVPA per week (MVPA = 6 METs) [42].

#### Quality of life

We measured participants’ quality of life in physical, mental, and social dimensions in the past four weeks using the Short Form 12 (SF-12) version (1) Version 1 was used since the service did not have a budget to pay the licence fee for version (2) The SF-12 is among the most frequently used multi-item health-related quality of life instruments. It is composed of eight multi-item scales (12 items) assessing physical function (2 items), role limitations due to physical health problems (2 items), bodily pain (1 item), general health (1 item), vitality (1 item), social functioning (1 item), role limitations due to emotional problems (2 items), and emotional well-being (2 items) [43]. These eight scales can be aggregated into the physical (PCS) and mental (MCS) component summary scores. The PCS and MCS scores were calculated by using the Hong Kong-derived weights for the eight scales. Higher scores represent better well-being.

#### Mood symptoms

The mood symptoms of the participants were measured using the 21-item Chinese version of the Depression, Anxiety, and Stress Scale [44]. The scale consists of three factors, namely depression, anxiety, and stress, and each of them has seven items. Respondents were asked to rate the frequency of each symptom in the past week on a 4-point Likert scale (0 = not at all, 1 = sometimes, 2 = often, and 3 = most of the time). The scores of each subscale are summed and multiplied by 2 to allow for the applicability of the cut-off scores of the long version. The validity and reliability of the scale were found to be satisfactory in both clinical and community samples [45]. Higher scores indicate more severe expressions of mood symptoms.

### Outcome measures

The outcome measures were the participants’ changes in cognitive functions, physical activity, quality of life, and mood symptoms before and after the intervention.

### Statistical analysis

Data were analysed using SPSS version 26 (IBM SPSS Statistics, New York, United States). All tests were two-sided with a significance level of *p* < .05. Descriptive statistics were calculated for baseline variables.

To examine if completers (n = 49) and non-completers (n = 38) of the programme were different in terms of their profiles, between-group comparison was performed using the independent two-sample t-test or Mann-Whitney Test for continuous variables and the chi-square test for categorical variables.

To test the hypotheses, the crude pre-post differences were examined using the paired-samples *t*-test or Wilcoxon signed-rank test (when the normality assumption was violated) for continuous variables, and the McNemar’s test for categorical variables was used to examine changes in outcome variables before and after the programme. The effect size (i.e., Cohen’s *d*, rank-biserial correlation *r*, or Cramer’s *V*) was calculated and presented for comparison. Multilevel models were used to examine the intervention effects, accounting for the individual variances that may affect the intervention effects. Participant’s subject code and background factors that were found to be correlated with the outcome measures (using correlation analysis) were put into the model as random factors, while the time point was put into the model as a fixed factor.

The Benjamini-Hochberg procedure with a false discovery rate (FDR) (q-value) of 5% was used [46] to handle the problem of multiple testing. All p-values presented were corrected.

## Results

### Participants profile

The profile of the participants is shown in Table 1. Of the 49 participants, most were female (87.8%), with an average age of 38.06 (SD = 13.15) years and an average education level of 13.18 (3.04) years. About one-tenth lived alone (9.2%). Resting heart rate and blood pressure were within the normal range in all participants. However, BMI and WHR indicated that they were slightly overweight (i.e., BMI 23.0–24.9 and WHR 0.80–0.85) (World Health Organisation, 2000). At baseline, participants averaged 1885.54 (SD = 2240.53) MET-min of walking and 631.56 (1399.91) MET-min of MVPA in the past week. Only 14.3% met the recommendation of PA for severe mental illnesses (i.e., ≥ 900 MET-min/week).

**Table 1:** Characteristics of participants (n = 87)

**Demographics**	
Age, mean (SD)	38.06 (13.15)
Female, n (%)	43 (87.8)
Living alone, n (%)	7 (14.3)
Year of education, n (%)	13.18 (3.04)
Employed, n (%)	19 (38.8)
**Physical fitness**	
Resting heart rate (bpm), mean (SD)	84.08 (11.67)
Resting SBP (mmHg), mean (SD)	111.16 (14.27)
Resting DBP (mmHg), mean (SD)	75.67 (8.73)
BMI, mean (SD)	24.01 (5.21)
WHR, mean (SD)	0.85 (0.06)
**Cognitive function**	
Digit span (forward), mean (SD)	12.37 (1.62)
Digit span (backward), mean (SD)	6.88 (2.86)
**Physical activity**	
Walking, MET-min/week, mean (SD)	1885.54 (2240.53)
MPA, MET-min/week, mean (SD)	305.91 (610.84)
VPA, MET-min/week, mean (SD)	340.00 (1046.65)
MVPA, MET-min/week, mean (SD)	631.56 (1399.91)
MVPA ≥ 900 MET-min/week, n (%)	7 (14.3)
**Quality of life**	
SF12 PCS, mean (SD)	44.24 (7.55)
SF12 MCS, mean (SD)	34.99 (13.19)
SF12 QoL, mean (SD)	39.61 (7.05)
**Mood symptoms**	
DASS Stress, mean (SD)	14.42 (9.01)
DASS Depression, mean (SD)	12.54 (9.20)
DASS Anxiety, mean (SD)	11.29 (7.73)
DASS Total, mean (SD)	38.25 (22.73)

***Note.*** SD = Standard deviation; n = frequency; bpm = beat per minute; SBP = Systolic blood pressure; DBP = Diastolic blood pressure; BMI = Body mass index; WHR = Waist-to-Hip ratio; MPA = moderate intensity physical activity; VPA = vigorous intensity physical activity; MVPA = moderate-to-vigorous intensity physical activity; SF-12 PCS = Short Form 12 Physical Component Summary score; SF-12 MCS = Short Form 12 Mental Component Summary score; DASS = Depression, Anxiety and Stress Scale

The SF-12 PCS (44.24 [SD = 7.55]) and MCS (34.99 [SD = 13.19]) indicated that the participant’s quality of life was worse than that of the general population of Hong Kong (48.6 [SD = 9.4] and 49.8 [SD = 9.6], respectively) [47]. The participants also experienced mild to moderate levels of mood symptoms. The participants averaged the forward digit span score at 12.37 (SD = 1.62) and the backward score at 6.88 (SD = 2.86). No statistically significant difference was observed between 49 programme completers and 38 non-completers (Supplementary Material 1).

### Effects of the FITMIND exercise programme

Correlation analysis found that participant’s employment was associated with higher digit span - backward score while BMI was negatively associated with SF-12 PCS scores (Supplementary Material 2). Changes in outcome measures among participants who completed the programme are shown in Table 2. A statistically significant improvement was observed in (on ascending order of *ES*): the number of participants achieving adequate MVPA, VPA, anxiety symptom severity, depressive symptom severity, stress level, and overall level of mood symptoms (*ES* ranged from 0.237 to 0.495). All differences remained robust after accounting for participants’ employment status and BMI through multilevel models. It’s noteworthy that participants’ MVPA became significant in the multilevel model analysis.

**Table 2:** Pre- and post-programme comparison

	N	Pre Mean (SD)	Post Mean (SD)	Crude test^a^			Multilevel models^b^
				ES	Power (1 - *β*)		B (95% CI)
**Cognitive function**							
Digit span (forward)	23	11.61 (1.88)	11.30 (2.53)	0.035	0.102		-0.54 (-1.61, 0.52)
Digit span (backward)	23	6.26 (2.73)	6.78 (3.34)	0.176	0.431		0.63 (-0.37, 1.62)
**Physical activity**							
Walking, MET-min/week	49	1885.54 (2240.53)	1782.00 (2041.32)	0.161	0.059		14.81 (-836.42, 866.03)
MPA, MET-min/week	49	305.91 (610.84)	347.56 (698.12)	0.002	0.062		51.62 (-229.63, 332.88)
VPA, MET-min/week	49	340.00 (1046.65)	733.04 (1278.83)	0.385*	0.334		394.77 (61.00, 728.55)*
MVPA, MET-min/week	49	631.56 (1399.91)	1073.04 (1632.8)	0.251	0.341		494.41 (25.84, 962.98)*
MVPA ≥ 900 MET-min/week, n (%)	49	7 (14.3)	17 (34.7)	0.237**	0.370		3.07 (1.13, 8.34)* ^*c*^
**Wellbeing**							
SF12 PCS	49	44.24 (7.55)	44.20 (9.37)	0.024	0.050		-0.72 (-2.85, 1.42)
SF12 MCS	49	34.99 (13.19)	37.32 (13.99)	0.164	0.197		2.59 (-1.73, 6.92)
**Mood symptoms**							
DASS Stress	49	14.42 (9.01)	11.14 (8.9)	0.446*	0.750		-3.51 (-6.17, -0.85)*
DASS Depression	49	12.54 (9.2)	8.94 (9.75)	0.417*	0.710		-3.90 (-6.66, -1.13)**
DASS Anxiety	49	11.29 (7.73)	8.61 (7.84)	0.409*	0.689		-2.83 (-4.99, -0.68)*
DASS Total	49	38.25 (22.73)	28.69 (24.9)	0.495**	0.786		-10.23 (-16.62, -3.84)**

***Note.*** ES = Effect size; SD = Standard deviation; n = frequency; MPA = moderate intensity physical activity; VPA = vigorous intensity physical activity; MVPA = moderate-to-vigorous intensity physical activity; SF-12 PCS = Short Form 12 Physical Component Score; SF-12 MCS = Short Form 12 Mental Component Score; SF-12 QoL = Short Form 12 Quality of Life Index; DASS = Depression, Anxiety and Stress Scale. * *p* < .01, * *p* < .05

^a^. Paired samples t-test or Wilcoxon signed-rank test was used

^b^. Multilevel models with participant’s subject code, employment status and baseline BMI were put into the model as random factors, while the time point was put into the model as a fixed factor

^c^. The statistic is the odds ratio computed

## Discussion

This is one of the few studies to demonstrate the potential efficacy of a community exercise programme for patients with psychosis. The comparison between participants who completed the programme and those who did not suggests that there is no discernible bias regarding programme compliance among the participants, even though the programme’s adherence needs more future efforts to improve. While previous exercise interventions were generally more intensive (ranging from 24 sessions in 12 weeks to 80 sessions in 16 weeks) [48], our 12-session exercise intervention, despite having fewer sessions, also demonstrated its beneficial effects in improving participant wellbeing in a number of ways. The results of this study suggest that the community exercise intervention is beneficial for patients with psychosis, particularly in terms of PA participation and improvement in mood symptoms. Typically, our programme illustrated how academic empirical findings could be implemented in practice.

On the forward digit span task, no improvement was observed, which may be due to the fact that participants’ baseline performance was within the normal range and significant improvement was not expected. Testing using the backward digit span test, an underpowered and non-significant improvement in participants’ working memory was observed. While exercise is a more effective intervention for enhancing working memory, with significant improvement in the exercise intervention group and deterioration in the control group [19, 49], a randomised controlled trial (RCT) will be needed to confirm the effect of the community FITMIND programme in improving working memory.

The results of this study suggest that participation in VPA and MVPA and adherence to international PA guidelines improved following the intervention. The higher MVPA level achieved after the programme suggests that the motivational barrier may have been easily overcome, as Firth et al. [16] suggested that achieving 107 min of MVPA participation (equivalent to 642 MET-min/week) was sufficient to achieve this objective. Coupled with the social support provided by the volunteers, which was consistently identified as an effective facilitator of exercise participation [50, 51], the potential longer-term effect of our FITMIND intervention appears promising and requires future studies to prove.

Consistent with previous research indicating that aerobic exercise and yoga can improve depressive symptoms [19, 52],our findings suggest that the FITMIND exercise programme may also improve stress and anxiety responses in participants. In this regard, a similar effect was also reported in patients with early psychosis engaging in yoga [53–55], whereas our study is the first to report a similar effect in community patients with psychosis. While moderate-to-vigorous-intensity physical activity (PA) stimulates the release of euphoria, which can improve mood [56],it may also engage sufficient attentional resources to suppress positive symptoms such as delusions and voices, resulting in less symptom distress.

Although it has been demonstrated that exercise is an effective adjunctive treatment for patients with psychosis in improving their quality of life, especially in the social and environmental dimensions [57], we did not observe this in the current 12-week FITMIND exercise programme. Nonetheless, it is possible that the sample size was insufficient to detect the effect; therefore, future research should be conducted to test this effect.

### Limitations

Our study has certain methodological limitations due to limited resources and the fact that the data were collected primarily to enhance services. First, the present investigation did not include a control group of patients. A randomised controlled trial is necessary in order to allow us to determine whether the benefits of the FITMIND exercise programme are linked to the absence of neurodegeneration and symptom alterations, as reported recently [58]. Second, providing data from the participants’ perspective is voluntary, which may lead to response bias. Third, PA levels were only measured subjectively and not objectively. Fourth, post-intervention cognitive function assessment refusal rates were high. Those who were willing to participate in an evaluation following the intervention may have exhibited greater improvement or less severe psychotic symptoms. Fifth, the majority of our sample was female (87.8%). Therefore, it is possible that the findings cannot be generalised to the entire psychosis population. However, this is not difficult to comprehend given that yoga is more popular among women than men. Sixth, the non-statistically significant improvement in other measures, such as walking, MPA, MVPA, well-being, and cognitive function, may have been attributable to the insufficient sample size for difference detection. It is also possible that the current intensity of exercise is insufficient to improve patients’ cardiovascular functioning, which was found to be a strong predictor of improved health outcomes in patients with psychosis [59, 60]; future research should include measurement of cardiovascular function. In spite of this, our findings provide additional evidence that a community exercise programme can improve PA participation and mood in psychosis patients. The programme also has the potential to enhance the participants’ general health and cognitive abilities. Last, the current evaluation approach was unable to delineate the separate effects of yoga and exercise, which represent different mechanisms of beneficial effects. Further research should investigate the independent effects of two interventions.

In conclusion, the FITMIND exercise programme, as a community-based intervention, demonstrated positive effects in terms of increasing PA participation and reducing negative emotional states in psychosis patients. Further systematic research is required to enhance programme participation and adherence, as well as examine the programme’s long-term benefits and cost-effectiveness.

## Electronic supplementary material

Below is the link to the electronic supplementary material.

Supplementary Material 1 The association between participant’s profile and outcome measures.

Supplementary Material 2 Comparison between programme completers and non-completers.

## Data Availability

The datasets used and/or analysed during the current study are available from the corresponding author on reasonable request.
