# Supplementary material for: Evaluating a pilot community-based FITMIND exercise programme for psychosis in Hong Kong
Source: BMC Psychiatry. 2023 May 31;23:385. doi: 10.1186/s12888-023-04901-x (PMC10234017; doi:10.1186/s12888-023-04901-x)
Supplement: Supplementary file 1 — Supplementary Material 1 The association between participant’s profile and outcome measures. [file 12888_2023_4901_MOESM1_ESM.docx]

**Supplementary material 1. The association between participant’s profile and outcome measures.**

|  |  | **Digit span (forward)** | **Digit span (backward)** | **Walking** | **MPA** | **VPA** | **MVPA** | **SF12**  **PCS** | **SF12 MCS** | **DASS Stress** | **DASS Depression** | **DASS Anxiety** | **DASS Total** |  | **MVPA ≥ 900 MET-min/week** | |  |
| --- | --- | --- | --- | --- | --- | --- | --- | --- | --- | --- | --- | --- | --- | --- | --- | --- | --- |
|  |  |  |  |  |  |  |  |  |  |  |  |  |  |  | **Yes** | **No** |  |
| **Age** | *r_s_* | -0.06 | -0.48 | -0.16 | 0.11 | 0.07 | 0.07 | -0.14 | 0.03 | -0.12 | 0.01 | -0.18 | -0.14 | Mean (SD) | 36.00 (12.43) | 39.79 (13.82) |  |
|  | *P* | 0.83 | 0.07 | 0.38 | 0.54 | 0.69 | 0.70 | 0.43 | 0.89 | 0.49 | 0.94 | 0.30 | 0.41 | *P^a^* | 0.40 | |  |
| **Year of education** | *r_s_* | 0.39 | 0.31 | 0.20 | -0.08 | -0.13 | -0.14 | 0.04 | 0.20 | -0.02 | -0.21 | -0.02 | -0.08 | Mean (SD) | 13.33 (2.44) | 13.08 (3.39) |  |
|  | *P* | 0.11 | 0.22 | 0.23 | 0.64 | 0.43 | 0.39 | 0.79 | 0.21 | 0.91 | 0.20 | 0.89 | 0.61 | *P^a^* | 0.80 | |  |
| **Resting heart rate** | *r_s_* | 0.26 | 0.22 | 0.06 | 0.14 | 0.07 | 0.12 | 0.01 | 0.05 | -0.16 | -0.24 | -0.10 | -0.15 | Mean (SD) | 84.35 (11.79) | 83.94 (11.79) |  |
|  | *P* | 0.24 | 0.31 | 0.70 | 0.34 | 0.66 | 0.43 | 0.92 | 0.72 | 0.26 | 0.10 | 0.47 | 0.30 | *P^a^* | 0.91 | |  |
| **Resting SBP** | *r_s_* | -0.25 | -0.23 | 0.19 | -0.06 | 0.01 | 0.01 | 0.00 | 0.07 | -0.15 | 0.07 | -0.06 | -0.02 | Mean (SD) | 110.35 (14.44) | 111.59 (14.39) |  |
|  | *P* | 0.24 | 0.28 | 0.21 | 0.72 | 0.93 | 0.95 | 0.98 | 0.62 | 0.31 | 0.64 | 0.67 | 0.89 | *P^a^* | 0.78 | |  |
| **Resting DBP** | *r_s_* | -0.10 | -0.14 | 0.16 | -0.03 | 0.09 | 0.09 | -0.09 | 0.15 | 0.01 | -0.03 | 0.02 | 0.03 | Mean (SD) | 77.24 (10.21) | 74.84 (7.88) |  |
|  | *P* | 0.66 | 0.52 | 0.30 | 0.86 | 0.55 | 0.55 | 0.52 | 0.29 | 0.95 | 0.82 | 0.92 | 0.86 | *P^a^* | 0.37 | |  |
| **BMI** | *r_s_* | -0.26 | -0.31 | 0.01 | -0.01 | 0.14 | 0.10 | -0.32 | 0.22 | 0.02 | -0.12 | -0.04 | -0.03 | Mean (SD) | 25.16 (4.94) | 23.37 (5.32) |  |
|  | *P* | 0.27 | 0.20 | 0.94 | 0.96 | 0.36 | 0.54 | **0.03** | 0.15 | 0.92 | 0.42 | 0.81 | 0.87 | *P^a^* | 0.27 | |  |
| **WHR** | *r_s_* | -0.02 | -0.13 | 0.08 | 0.08 | -0.07 | -0.03 | -0.10 | 0.12 | -0.23 | -0.02 | -0.18 | -0.17 | Mean (SD) | 0.84 (0.07) | 0.85 (0.05) |  |
|  | *P* | 0.94 | 0.59 | 0.62 | 0.60 | 0.66 | 0.86 | 0.51 | 0.42 | 0.12 | 0.89 | 0.23 | 0.26 | *P^a^* | 0.52 | |  |
| **Gender** |  |  |  |  |  |  |  |  |  |  |  |  |  |  |  |  |  |
| **Male** | Mean (SD) | 12.00 (0.71) | 8.80 (3.27) | 2629.00 (1897.05) | 200.00 (319.00) | 373.33 (587.15) | 573.33 (737.42) | 49.68 (3.23) | 35.78 (17.46) | 9.33 (6.41) | 11.00 (14.35) | 8.67 (9.61) | 29.00 (29.39) | N (%) | 2 (11.8) | 4 (12.5) |  |
| **Female** | Mean (SD) | 11.11 (2.83) | 6.22 (3.23) | 1651.69 (2054.43) | 370.26 (739.57) | 787.00 (1349.05) | 1148 (1721.14) | 43.43 (9.71) | 37.53 (13.67) | 11.40 (9.22) | 8.65 (9.13) | 8.60 (7.70) | 28.65 (24.61) |  | 15 (88.2) | 28 (87.5) |  |
|  | *P^a^* | 0.50 | 0.13 | 0.28 | 0.58 | 0.47 | 0.43 | 0.13 | 0.78 | 0.60 | 0.59 | 0.99 | 0.98 | *P^b^* | 0.94 | |  |
| **Living status** |  |  |  |  |  |  |  |  |  |  |  |  |  |  |  |  |  |
| **Living alone** | Mean (SD) | 9.00 (4.36) | 3.67 (1.53) | 858.00 (961.26) | 243.33 (210.30) | 380.00 (445.24) | 623.33 (644.29) | 41.18 (10.43) | 37.38 (12.50) | 13.43 (12.20) | 10.57 (13.4) | 10.86 (13.36) | 34.86 (38.73) | N (%) | 2 (11.8) | 5 (15.6) |  |
| **Others** | Mean (SD) | 11.65 (2.11) | 7.25 (3.31) | 1924.15 (2132.55) | 363.59 (746.01) | 786.00 (1356.30) | 1140.50 (1728.35) | 44.70 (9.23) | 37.31 (14.36) | 10.76 (8.36) | 8.67 (9.19) | 8.24 (6.70) | 27.67 (22.33) |  | 15 (88.2) | 27 (84.4) |  |
|  | *P^a^* | 0.91 | 0.08 | 0.24 | 0.70 | 0.48 | 0.48 | 0.36 | 0.99 | 0.47 | 0.64 | 0.42 | 0.49 | *P^b^* | 0.71 | |  |
| **Employment status** |  |  |  |  |  |  |  |  |  |  |  |  |  |  |  |  |  |
| **Employed** | Mean (SD) | 11.33 (3.00) | **8.56 (3.81)** | 1485.00 (1439.73) | 241.11 (346.03) | 475.56 (792.66) | 716.67 (982.30) | 45.10 (8.13) | 38.10 (13.75) | 13.47 (10.79) | 8.95 (11.91) | 8.84 (8.70) | 31.26 (30.52) | N (%) | 6 (35.3) | 13 (40.6) |  |
| **Others** | Mean (SD) | 11.29 (2.30) | **5.64 (2.53)** | 1980.00 (2365.32) | 418.52 (856.40) | 898.57 (1502.49) | 1302.14 (1922.70) | 43.62 (10.18) | 36.83 (14.35) | 9.67 (7.28) | 8.93 (8.32) | 8.47 (7.40) | 27.07 (20.99) |  | 11 (64.7) | 19 (59.4) |  |
|  | *P^a^* | 0.97 | **0.04** | 0.43 | 0.41 | 0.28 | 0.24 | 0.60 | 0.76 | 0.15 | >0.99 | 0.87 | 0.57 | *P^b^* | 0.72 | |  |
| *r_s_* = Spearman correlation coefficient. SBP = Systolic blood pressure; DBP = Diastolic blood pressure; BMI = Body mass index; WHR = Waist-to-Hip ratio; MPA = moderate intensity physical activity; VPA = vigorous intensity physical activity; MVPA = moderate-to-vigorous intensity physical activity; SF-12 PCS = Short Form 12 Physical Component Summary score; SF-12 MCS = Short Form 12 Mental Component Summary score; DASS = Depression, Anxiety and Stress Scale.  a. Between-group comparison using independent samples t-test.  b. Between-group comparison using Chi-square test. | | | | | | | | | | | | | | | | | |
