# Supplementary material for: Evaluating a pilot community-based FITMIND exercise programme for psychosis in Hong Kong
Source: BMC Psychiatry. 2023 May 31;23:385. doi: 10.1186/s12888-023-04901-x (PMC10234017; doi:10.1186/s12888-023-04901-x)
Supplement: Supplementary file 2 — Supplementary Material 2 Comparison between programme completers and non-completers. [file 12888_2023_4901_MOESM2_ESM.docx]

# Supplementary material 2

## **Comparison between programme completers and non-completers.**

|  | **Completers**  **(n = 49)** | **Non-completers**  **(n = 38)** | **ES** |
| --- | --- | --- | --- |
| **Demographics** |  |  |  |
| Age, mean (SD | 38.06 (13.15) | 31.46 (10.73) | 0.256 |
| Female, n (%) | 43 (87.8) | 35 (92.1) | 0.071 |
| Living alone, n (%) | 7 (14.3) | 1 (2.6) | 0.200 |
| Year of education, n (%) | 13.18 (3.04) | 13.54 (2.63) | 0.041 |
| Employed, n (%) | 19 (38.8) | 14 (36.8) | 0.047 |
| **Physical fitness/activity** |  |  |  |
| Resting heart rate (bpm), mean (SD) | 84.08 (11.67) | 88.32 (14.29) | 0.169 |
| Resting SBP (mmHg), mean (SD) | 111.16 (14.27) | 108.79 (16.96) | 0.091 |
| Resting DBP (mmHg), mean (SD) | 75.67 (8.73) | 73.13 (11.33) | 0.112 |
| BMI, mean (SD) | 24.01 (5.21) | 24.06 (4.92) | 0.009 |
| WHR, mean (SD) | 0.85 (0.06) | 0.85 (0.07) | 0.007 |
| **Cognitive function** |  |  |  |
| Digit span (forward), mean (SD) | 12.37 (1.62) | 11.55 (2.40) | 0.145 |
| Digit span (backward), mean (SD) | 6.88 (2.86) | 6.51 (2.79) | 0.047 |
| **Physical activity level** |  |  |  |
| Walking, MET-min/week, mean (SD) | 1885.54 (2240.53) | 1907.87 (2616.7) | 0.022 |
| MPA, MET-min/week, mean (SD) | 305.91 (610.84) | 356.57 (847.27) | 0.018 |
| VPA, MET-min/week, mean (SD) | 340.00 (1046.65) | 1141.62 (4438.61) | 0.057 |
| MVPA, MET-min/week, mean (SD) | 631.56 (1399.91) | 1440.00 (4487.99) | 0.038 |
| MVPA ≧900 MET-min/week, n (%) | 7 (14.3) | 12 (31.6) | 0.190 |
| **Wellbeing** |  |  |  |
| SF12 PCS, mean (SD) | 44.24 (7.55) | 47.40 (9.61) | 0.193 |
| SF12 MCS, mean (SD) | 34.99 (13.19) | 30.28 (14.25) | 0.184 |
| SF12 QoL, mean (SD) | 39.61 (7.05) | 38.84 (8.12) | 0.096 |
| **Mood symptoms** |  |  |  |
| DASS Stress, mean (SD) | 14.42 (9.01) | 17.35 (10.90) | 0.142 |
| DASS Depression, mean (SD) | 12.54 (9.20) | 15.68 (11.22) | 0.127 |
| DASS Anxiety, mean (SD) | 11.29 (7.73) | 13.78 (9.86) | 0.128 |
| DASS Total, mean (SD) | 38.25 (22.73) | 46.81 (29.41) | 0.149 |
| ***Note.*** ES = Effect size; SD = Standard deviation; n = frequency; bpm = beat per minute; SBP = Systolic blood pressure; DBP = Diastolic blood pressure; BMI = Body mass index; WHR = Waist-to-Hip ratio; MPA = moderate intensity physical activity; VPA = vigorous intensity physical activity; MVPA = moderate-to-vigorous intensity physical activity; SF-12 PCS = Short Form 12 Physical Component Summary score; SF-12 MCS = Short Form 12 Mental Component Summary score; SF-12 QoL = Short Form 12 Quality of Life Index; DASS = Depression, Anxiety and Stress Scale. | | | |
